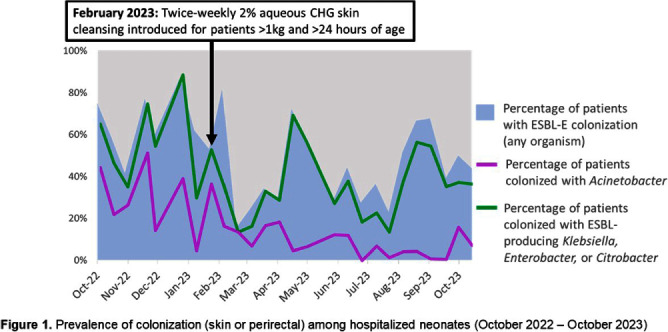# Temporal decreases in pathogen colonization and infection among hospitalized neonates following routine skin antisepsis with chlorhexidine gluconate: Botswana 2022 – 2023

**DOI:** 10.1017/ash.2024.111

**Published:** 2024-09-16

**Authors:** Chimwemwe Tembo, Jonathan Strysko, Boingotlo Gopolang, Tshiamo Zankere, Teresia Gatonye, Tlhalefo Dudu Ntereke, Kgomotso Kgomanyane, Janet Thubuka, Britt Nakstad, Susan Coffin, Carolyn McGann, Corrado Cancedda, Ebbing Lautenbach, Naledi Betsi Mannathoko, Melissa Richard-Greenblatt, David Goldfarb, Ashley Styczynski, Gemma Parra, Rachel Smith, Kagiso Mochankana, Neo Mogotsi, Tapoloso Keatholetswe

**Affiliations:** Botswana-University of Pennsylvania partnership; Children’s Hospital of Philadelphia; Botswana UPenn Partnership; Botswana UPenn; Ministry of Health and Wellness-Botswana; Dept Paediatric and Adolescent Health, Univ Botswana; University of Pennsylvania; Perelman School of Medicine; University of Botswana; Hospital for Sick Children; BC Children’s Hospital; Centers for Disease Control and Prevention; University of Arizona; Nurses & midwifery association of Botswana

## Abstract

**Background:** Multidrug-resistant Gram-negative bacteria are a major cause of sepsis among hospitalized neonates globally. Aqueous chlorhexidine gluconate (CHG) skin antisepsis has been shown to be safe for use in infants; however, its sustained effectiveness in preventing Gram-negative pathogen colonization, bloodstream infection (BSI), and mortality is unclear. **Methods:** We conducted a period prevalence survey, with 26 sampling events over 12 months (18 October 2022 – 31 October 2023) at a 33-bed neonatal unit in a tertiary public hospital in Botswana where ESBL-producing Klebsiella pneumoniae and carbapenem-resistant Acinetobacter baumannii are leading causes of BSI. Perirectal and periumbilical skin swabs were collected every two weeks from all inpatients. Swabs were inoculated onto chromogenic media selective and differential for extended-spectrum beta-lactamase producing Enterobacterales (ESBL-E) and Acinetobacter spp. (CHROMagar™ ESBL, Acinetobacter). Colonization status was determined based on culture growth and colony morphology. Contemporaneous data on all-cause mortality and BSI were abstracted from routine surveillance records. Pre- and post-CHG prevalences were compared using a simple Chi-square test. During the surveillance period, an outbreak of K. pneumoniae linked to contaminated multi-use vials was detected, thus BSIs and deaths during the outbreak period (2 February–6 April, 2023) were excluded. In February 2023, the hospital infection prevention and control (IPC) team introduced twice-weekly whole-body cleansing with commercially available 2% aqueous CHG, performed by caregivers and healthcare workers on neonates >24 hours old and weighing ≥1 kg until discharge. **Results:** There were significant decreases in ESBL-E and Acinetobacter skin and perirectal colonization following the CHG intervention (Table 1; Figure 1). After the CHG intervention, the incidence of Acinetobacter BSIs declined significantly and there was a trend toward a decline in other BSIs and mortality. No adverse events associated with CHG were reported. **Conclusions:** Twice-weekly CHG application was temporally associated with significant reductions in neonatal ESBL-E and Actinetobacter skin and perirectal colonization and Acinetobacter BSI. This analysis was limited by a short pre-intervention surveillance period and thus may have been influenced by confounders such as seasonality, and intensified IPC efforts following the outbreak. Analysis of the routine CHG use in other settings and over longer surveillance periods are needed to better understand its effectiveness as an IPC strategy in settings where neonatal sepsis incidence is high. Table 1. Colonization prevalence, BSI incidence, and mortality surrounding introduction of CHG skin cleansing in a neonatal unit, 18 October 2022 – 31 October 2023.

**Figure:**
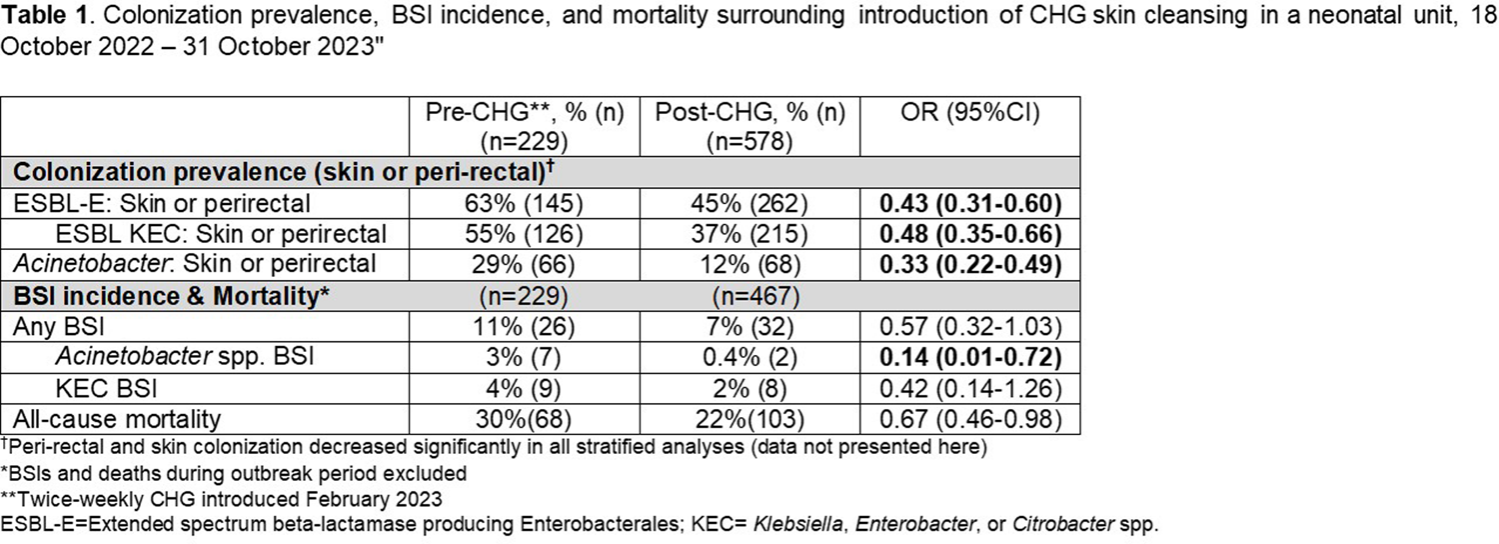

**Figure:**